# 肺癌患者术后饮食种类与方案对其住院舒适度和康复的影响

**DOI:** 10.3779/j.issn.1009-3419.2024.102.21

**Published:** 2024-06-20

**Authors:** Xue YANG, Lingling ZUO, Ziyi ZHAO, Li TU, Qilian WANG, Munai JIAGA, Hongjuan LI, Guowei CHE

**Affiliations:** ^1^610041 成都，四川大学华西医院肺癌中心/肺癌研究所（杨雪，左玲玲，赵梓邑，涂梨，王启莲，甲呷木乃，李洪娟，车国卫）; ^1^Lung Cancer Center, West China Hospital; ^2^华西护理学院（杨雪，左玲玲，王启莲，甲呷木乃，李洪娟）; ^2^West China School of Nursing; ^3^华西医院肿瘤中心（涂梨）; ^3^Department of Thoracic Surgery, West China Hospital; ^4^华西医院胸外科（赵梓邑，车国卫）; ^4^Department of Medical Oncology, Cancer Center, West China Hospital, Sichuan University, Chengdu 610041, China

**Keywords:** 肺肿瘤, 中链甘油三酯饮食, 膳食满意度, 住院舒适度, Lung neoplasms, Medium chain triglyceride diet, Dietary satisfaction, Hospitalization comfort

## Abstract

**背景与目的** 肺癌术后合理、规范的饮食方案与流程有助于患者快速康复。本研究旨在通过优化以中链甘油三酯（medium chain triglyceride, MCT）为主的饮食方案与流程，探讨其对肺癌患者术后康复的临床优势。**方法** 收集2023年10月至2023年12月在四川大学华西医院肺癌中心行肺癌手术的患者共156例，其中MCT组76例和常规饮食（routine diet, RD）组80例，分析比较两组患者术后临床症状、生化指标、术后住院时间及费用、膳食满意度与住院舒适度等指标的差异。**结果** 术后肛门排气时间MCT组为24.00（9.75, 36.97）h，显著短于RD组的28.50（24.00, 48.00）h（P<0.001）；MCT组头晕和恶心呕吐发生率分别为18.42%和6.58%，显著低于RD组的51.25%和31.25%（P<0.001, P<0.001）；MCT组住院舒适度评分为（16.74±1.70）分，显著高于RD组的（14.83±2.34）分（P=0.016）；MCT组平均住院费用为（39,701.82±8105.47）¥，显著低于RD组的（44,511.79±9593.19）¥（P=0.007）。**结论** 肺癌患者术后以MCT为主的饮食方案与流程优化有助于促进患者胃肠功能的恢复，提高住院舒适度并加速术后康复。

## Abstract (en)

**Background and objective** A reasonable and standardized dietary plan and procedure can help patients recovering quickly from lung cancer surgery. The aim of this study is to optimize the diet plan and procedure mainly based on medium chain triglyceride (MCT) diet and explore its clinical advantages for postoperative lung cancer patients. **Methods** From October 2023 to December 2023, a total of 156 patients were collected, who underwent lung cancer surgery in Lung Cancer Center, West China Hospital of Sichuan University. The patients were randomized into MCT group (76 cases) and routine diet (RD) group (80 cases). Clinical symptoms, biochemical index, postoperative hospitalization time and cost, dietary satisfaction and hospitalization comfort between the two groups were analyzed. **Results** The mean anus exhausting time in MCT group [24.00 (9.75, 36.97) h] was significantly shorter than that in RD group [28.50 (24.00, 48.00) h] (P<0.001). And the incidence of dizziness (18.42%), nausea and vomiting (6.58%) in MCT group were remarkably lower than those in RD group (51.25%, 31.25%) (P<0.001). Hospitalization comfort score in MCT group [(16.74±1.70)] was significantly higher than that in RD group [(14.83±2.34)] (P=0.016). Meanwhile, the average hospitalization cost in MCT group [(39,701.82±8105.47)¥] showed an obvious decrease compared with RD group [(44,511.79±9593.19)¥] (P=0.007). **Conclusion** Optimizing the dietary plan and procedure mainly based on MCT diet for postoperative lung cancer patients can help the recovery of gastrointestinal function and improve hospitalization comfort, which promoted overall postoperative rehabilitation of patients with lung cancer surgery.

肺癌胸腔镜外科技术的普及及快速康复理念的推广，使肺癌患者术后早期康复得以实现，极大地改善了肺癌患者的临床诊疗体验^[1,2]^，围手术期营养管理是快速康复理念的重要组成部分^[3]^。传统的诊疗理念仍采用术后常规禁食禁饮后轻流质转常规饮食（routine diet, RD）的理念，但快速康复理念认为术后应更早期开始肠内营养，同时应用易于消化吸收的营养制剂及餐食，促进患者术后胃肠道功能的恢复^[4]^。研究^[5,6]^表明，肺癌术后患者全麻清醒2 h即开始进食全流质饮食可明显降低术后恶心呕吐的发生率，且不会增加肺部并发症的发生率。术后进食以长链脂肪酸（long chain triglyceride, LCT）为主的饮食，可能导致乳糜胸及胃肠功能障碍^[7]^；而以中链甘油三酯（medium chain triglyceride, MCT）为主的饮食，分子量小、易于吸收，能够快速而有效地被氧化供能，减轻肠道负担，且经肠摄入的MCT不经淋巴系统转运，可减少手术患者淋巴管瘘导致的脂肪丢失和引流液产生^[8]^。杜娜等^[9]^开展的有关MCT饮食的前瞻性随机研究亦发现肺癌术后短期应用MCT饮食有助于患者胃肠功能的快速恢复，缩短术后平均住院时间。但是，目前我国肺癌术后患者的早期饮食方案尚无统一标准。四川大学华西医院肺癌中心在临床实践中不断推行加速康复理念，联合肺癌多学科诊疗团队制定了肺癌术后以MCT饮食为主的全流程方案。本研究旨在通过比较以MCT饮食为主的流程方案与传统禁食禁饮后流质饮食的RD方案的效果差异，探讨促进肺癌术后患者胃肠功能恢复及整体康复的更优饮食方案及流程。

## 1 资料与方法

### 1.1 研究对象

收集2023年10月至2023年12月于四川大学华西医院肺癌中心接受肺癌根治术的180例患者的资料。纳入标准：（1）年龄18-85岁；（2）病理学检查确诊肺恶性肿瘤；（3）手术方式为肺段/肺叶切除术+系统淋巴结清扫术；（4）患者临床资料完整，签署知情同意书。排除标准：（1）病理学检查诊断为转移性肺癌；（2）未签署知情同意书；（3）术前诊断为营养不良或术后在重症监护室监护超过48 h；（4）发生非计划二次手术；（5）胸腔镜中转开胸；（6）病例资料不完整。该研究经四川大学华西医院生物医学伦理审查委员会批准[批准号：2023年审（1205）号]。

对初始纳入的患者进行随机数字化分组，分为MCT组与RD组；排除病理良性及资料不全患者24例，最终纳入患者156例；其中MCT组76例，RD组80例。术后分期采用国际抗癌联盟（Union for International Cancer Control, UICC）（第8版）肺癌分期标准。患者基线临床特征间差异无统计学意义（P>0.05）（表1）。

**表1:** MCT组与RD组肺癌患者间临床病理生理特征比较

Index	MCT group (n=76)	RD group (n=80)	P
Age (yr)	49.89±11.15	51.30±13.14	0.059
Gender			0.978
Male	22 (28.95%)	23 (28.75%)	
Female	54 (71.05%)	57 (71.25%)	
Smoking			0.270
Yes	11 (14.47%)	17 (21.25%)	
No	65 (85.53%)	63 (78.75%)	
Comorbidity			
Diabetes	2 (2.63%)	4 (5.00%)	0.731
Hypertension	11 (14.47%)	10 (12.50%)	0.747
pTNM stage			0.898
I	73 (96.05%)	75 (93.75%)	
II	1 (1.32%)	1 (1.25%)	
III	1 (1.32%)	2 (2.50%)	
IV	1 (1.32%)	2 (2.50%)	
BMI (kg/m^2^)	22.10±2.72	22.10±2.52	0.221
Chylothorax	0 (0.00%)	0 (0.00%)	>0.999
Surgical time (min)	84.71±29.19	75.78±29.87	0.869

MCT: medium chain triglyceride; RD: routine diet; pTNM: pathological tumor-node-metastasis; BMI: body mass index.

### 1.2 手术方法及引流管处理方案

手术方法：采用单向式胸腔镜手术法^[10]^。手术为三孔入路，观察孔位于腋中线第7肋间，主操作孔位于腋前线第3肋间或第4肋间，副操作孔位于腋后线与肩胛线之间第9肋间。左侧须清扫第5、6、7、8、9、10组淋巴结，右侧须清扫第2、4、7、8、9、10组淋巴结^[11]^。所有患者术后均用单引流管，将引流管从观察孔置入胸腔，从后胸壁放至胸顶，线结固定并留置预留线^[12]^，术后采用相同的水封引流瓶，均不加用负压吸引。

### 1.3 术后饮食管理

MCT组：术前8 h禁食和术前2 h禁水；术后2 h清醒后口服100 mL温水；术后2-3 h饮用清流质粉[清流质粉主要成分：热量（192.5 kcal）、碳水化合物（47.5 g）]，兑温水200 mL；术后4-8 h口服肠内营养粉50 g/包[肠内营养主要成分：热量（206.2 kcal）、蛋白质（14.5 g）、脂肪（3.43 g）、碳水化合物（28.4 g）、钠（132.5 mg）、钾（190 mg）、钙（146 mg）]，兑温水200 mL，服用2次；术后8 h到出院，按需MCT饮食，可喝水，进食水果；术后第3天恢复正常饮食。RDG组：采用常规饮食护理，术前晚禁食禁饮，术后禁食禁饮4 h；术后6-8 h患者神志清楚后可口服适量温水；无恶心、呕吐不适者，术后6-8 h可进清流质饮食；术后第1天可正常饮食（表2）。

**表2:** 两组患者术后的饮食方案与流程

Time	MCT group (n=76)	RD group (n=80)
PP12 h		Fasting and abstinence
PP2 h	Fasting and abstinence	Fasting and abstinence
POP2 h	Water	Fasting and abstinence
POP4-6 h	Clear liquid powder	Water
POP8 h	Enternal nutrition powder	Water
POP12 h	MCT	Routine diet
POP12 h--Charge	MCT	Routine diet

PP: pre-operation; POP: post-operation.

对照组（RD组）：采用RD，术后禁食禁饮4 h；术后4-6 h患者神志清楚后可口服适量温水；无恶心、呕吐不适者，术后6-8 h可进清流质饮食；术后第1天正常饮食。

### 1.4 术后处理

术后鼓励患者咳嗽；指导患者进行呼吸功能锻炼及早期下床活动。术后第1天行胸部正侧位照片或者胸部平扫计算机断层扫描（computed tomography, CT）检查，若无漏气且每天引流量小于300 mL，肺已复张、无血性或乳糜性引流液则拔除引流管^[13]^。术后两组患者均采用镇痛泵（1 mL/h），必要时应用阿片类止痛药（地佐辛），拔除引流管并停用镇痛泵。

### 1.5 观察指标

#### 1.5.1 症状及严重程度评估

应用肺癌患者术后症状量表^[14]^进行评估。该量表Cronbach’s α系数为0.964，内容效度指数为0.954。

#### 1.5.2 乳糜胸

诊断标准：乳糜试验（+）且每天引流量大于500 mL。

#### 1.5.3 胸腔引流管留置时间及引流量

引流管留置时间为从安置到拔除时间；术后胸腔引流量为从胸腔引流管安置到拔除时的总引流量。

#### 1.5.4 术后主要症状

肛门排气时间（从手术结束到患者自诉肛门排气时间）、头晕、恶心、呕吐及腹胀腹泻、咳嗽及疲劳气促。

#### 1.5.5 术后生化指标

术后第3天采空腹静脉血，检测肝、肾功能等指标，包括血白蛋白（albumin, ALB）、肌酐（creatinine, Cr）、谷草转氨酶（aspartate aminotransferase, AST）及谷丙转氨酶（alanine aminotransferase, ALT）。

#### 1.5.6 术后住院日及住院总费用

术后住院日指术后第1天到出院当天时间；住院总费用指住院期间所产生的费用。

#### 1.5.7 住院膳食满意度评价

采用自制量表对膳食份量、种类、口味、消化进行评价，各维度10分，满分40分；出院当天发放满意度调查问卷，专人指导患者填写。

#### 1.5.8 住院舒适度

采用胸腔镜术后患者舒适度量表^[15]^进行调查。该量表Cronbach’s α系数为0.801，量表内容效度指数为0.97。评估项目包括进食、穿衣、下床活动、如厕、咳嗽、疼痛、睡眠、疲劳、负性情绪、头晕10个维度，各维度采用Likert 3级计分法，总分17-20分表示舒适，总分5分以下表示非常不舒适。出院当天发放舒适度调查问卷，专人指导病员填写。

### 1.6 统计学处理

采用SPSS 26.0软件包进行统计分析，计数资料采用实际例数及百分比表示，正态分布的计量资料采用均数±标准差表示，比较采用两独立样本t检验；非正态分布计量资料采用中位数和四分位数间距表示，比较采用两独立样本的秩和检验（Mann-Whitney U检验）；计数资料采用χ^2^检验或确切概率法进行比较。P<0.05为差异有统计学意义。

## 2 结果

### 2.1 MCT组与RD组肺癌患者的膳食满意度、住院舒适度及住院费用分析

饮食方案份量评分、种类评分、口味评分在MCT组显著高于RD组，组间比较有统计学差异（P<0.001, P=0.010, P=0.013）；住院舒适度评分在MCT组显著高于RD组，组间比较有统计学差异（P=0.016）；平均住院费用在MCT组显著低于RD组，组间比较有统计学差异（P=0.007）。但是，MCT组患者膳食满意度总评分和RD组比较无统计学差异（P=0.491）（表3）。

**表3:** MCT组与RD组肺癌患者膳食满意度、住院舒适度及住院费用的调查结果比较

Dietary evaluation	MCT group (n=76)	RD group (n=80)	P
Portion rating	9.75±0.98	8.93±1.49	<0.001
Category rating	8.03±1.87	6.01±1.72	0.010
Taste rating	8.35±1.71	5.93±1.68	0.013
Digestibility rating	8.93±1.45	6.08±2.00	0.491
Total comfort score	16.74±1.70	14.83±2.34	0.016
Average hospital cost (¥)	39,701.82±8105.47	44,511.79±9593.19	0.007

### 2.2 不同饮食方案对肺癌患者术后胃肠功能及胸腔引流量的影响

术后MCT组与RD组患者的肛门排气时间、胸腔引流量及胸腔引流管留置时间的差异均具有统计学意义（P<0.001, P<0.001, P<0.001）（表4）。

**表4:** MCT组与RD组患者术后胃肠功能及胸腔引流量时间情况比较

Index	MCT group (n=76)	RD group (n=80)	P
Anus exhaust time (h)	24.00 (9.75, 36.97)	28.50 (24.00，48.00)	<0.001
Chest drainage volume (mL)	55.00 (15, 120)	200.00 (100, 375)	<0.001
Chest drainage time (h)	28.00 (24, 45)	47.00 (23, 150)	<0.001

### 2.3 不同饮食方案对肺癌患者术后主要临床症状及术后住院时间的影响

MCT组头晕和恶心呕吐发生率显著低于RD组，咳嗽发生率高于RD组，两组间比较有统计学差异（P<0.001, P<0.001, P=0.001）；MCT组腹胀、腹泻、疲劳气促、术后住院时间的发生率和RD组比较均无统计学差异（P=0.073, P=0.530, P=0.221, P=0.704）（表5）。

**表5:** MCT组与RD组患者术后临床症状的比较

Index	MCT group (n=76)	RD group (n=80)	P
Dizziness	14 (18.42%)	41 (51.25%)	<0.001
Nausea and vomiting	5 (6.58%)	25 (31.25%)	<0.001
Abdominal distension	6 (7.89%)	14 (17.5%)	0.073
Diarrhea	2 (2.63%)	1 (1.25%)	0.530
Fatigue and dyspnea	73 (96.05%)	73 (91.25%)	0.221
Cough	70 (92.11%)	58 (72.50%)	0.001
Post-hospitalization time (d)	2.86±1.58	2.91±1.12	0.704

### 2.4 不同饮食方案对肺癌患者白蛋白水平及肝肾功能的影响

MCT组患者术后血白蛋白（albumin, ALB）低于RD组，组间比较无统计学差异（P=0.130）；MCT组患者术后血清肌酐（creatinine, Cr）显著高于RD组，组间比较有统计学差异（P=0.041）；MCT组与RD组患者术后血谷草转氨酶（aspartate aminotransferase, AST）与血谷丙转氨酶（alanine aminotransferase, ALT）比较无统计学差异（P=0.097, P=0.179）（表6）。

**表6:** MCT组与RD组肺癌患者间术后白蛋白及肝肾功能指标比较

Index	MCT group (n=76)	RD group (n=80)	P
Albumin (g/L)	41.16±5.11	41.44±3.26	0.130
Creatinine (mmol/L)	66.24±21.14	62.51±16.61	0.041
AST (U/L)	24.20±7.11	24.64±12.15	0.097
ALT (U/L)	18.79±8.28	18.15±9.24	0.179

AST: aspartate aminotransferase; ALT: alanine aminotransferase.

## 3 讨论

肺癌手术操作会导致机体产生应激反应，机体分泌大量如肿瘤坏死因子α（tumor necrosis factor α, TNF‐α）、白细胞介素6（interleukin 6, IL‐6）、白细胞介素8（interleukin 8, IL‐8）、血栓素A（thromboxane A, TXA）等炎性细胞因子^[16,17]^，使患者免疫功能下降；加上手术创伤、麻醉、术中出血、术后恶心及食欲较差等因素的影响，肺癌患者更容易出现术后营养不良^[18,19]^。患者术后应尽早恢复经口摄食或给予口服补液盐溶液（oral rehydration solution, ORS）^[20]^，可以有效改善患者的部分临床症状和住院感受。合适和合理的饮食种类有助于促进机体对于营养的吸收，减轻肠道负担，促进肠道功能早期恢复。已有的研究^[21,22]^表明：MCT饮食具有以下特点：（1）分子量小，在生物体内溶解度更高，易于吸收；（2）不需肉碱存在，很快通过线粒体膜，迅速而有效地被氧化供能；（3）直接以脂肪酸形式由门静脉进入肝脏，在肝脏内迅速高效地被分解产生能量；（4）轻度降低胆固醇吸收，并减慢肝内合成。MCT饮食的以上特点使其更加适合作为肺癌患者术后的饮食。

四川大学华西医院肺癌中心在长期的临床实践过程中，不断优化MCT饮食为主的饮食方案和流程：术后2 h即给予患者口服温水，若无恶心呕吐等副反应的发生，即给予患者清流质粉及肠内营养粉，为患者提供热能、蛋白质、碳水化合物及微量元素；术后8 h到出院即给予患者营养科配制的以优质植物蛋白为主的MCT饮食。本研究结果发现优化后的进食时间为2 h，这一举措改善了肺癌患者术后的胃肠功能，缩短了术后肛门排气时间，减少了术后胸腔引流量；同时，MCT组患者术后白蛋白指标较RD组也有所改善。本研究打破术后6 h饮食传统，通过饮食流程优化，即术后2 h即开始饮水并逐步过渡到MCT饮食，与营养科合作，对患者术后饮食进行早期的专业化的营养管理，选择营养科统一配制的膳食即MCT饮食，促进患者胃肠功能恢复，加速患者早期康复。

此外，在临床实践中，我们发现患者家属准备患者术后饮食存在一定的困惑，优化的MCT饮食可以减轻患者家属围手术期的照护负担，提升患者的膳食满意度及住院舒适度。但是，在优化MCT饮食的实施过程中也存在一些问题，包括患者的依从性和医护人员的支持力度。加速康复外科理念要求对外科术后的患者进行全方位的管理需要多学科、亚专业团队的协作，同时也需进一步加强医护团队之间的合作^[23]^。创建舒适化病房，改善患者满意度及舒适度是优质护理模式的应有之意^[24]^。未来将进一步加强医疗与护理团队的医护一体化管理，加强团队协作，加强医护人员对患者的宣教，如讲座及公众号进行科普推广，探索肺癌术后简单易行的标准化进食模式，从而改善患者术后症状，减少患者并发症的发生，加速患者康复。

综上所述，优化MCT饮食能够促进肺癌术后患者胃肠道功能的恢复，减少患者的胸腔引流量，一定程度上改善患者术后营养状况，提高患者膳食满意度及住院舒适度，加速患者术后康复。但本研究为单中心研究，不能排除结果的偏倚，希望未来可进一步扩大研究范围，对加速康复外科理念进行证实和推广。
